# Telemedicine and the European Health Data Space: a new paradigm for healthcare in the EU

**DOI:** 10.3389/fdgth.2025.1713758

**Published:** 2026-01-09

**Authors:** Margarida Carradinha, Vanessa I. S. Mendes, Rui Pedro Moura, Nuno P. Silva, Laura Rocha, João Gonçalves, Inês Antunes, Eirini Schiza, Constantinos S. Pattichis, Alberto Zanini, Vanja Pajić, Cátia S. Pinto

**Affiliations:** 1 Global Digital Health and International Affairs Unit, SPMS - Shared Services of the Ministry of Health, E. P. E., Lisbon, Portugal; 2 Planning, Architecture, Compliance and Engineering Unit (UPACE), SPMS - Shared Services of the Ministry of Health, E. P. E., Lisbon, Portugal; 3 Department of Computer Science and Biomedical Engineering Research Center (BERC), University of Cyprus, Nicosia, Cyprus; 4 Maggioli SpA, Santarcangelo di Romagna, Italy; 5 Independent eHealth Interoperability Consultant, Zagreb, Croatia

**Keywords:** artificial intelligence, cross-border, digital health, European Health Data Space, MyHealth@EU, remote care, telemedicine

## Abstract

**Background:**

Telemedicine has emerged as a transformative tool for remote healthcare, taking advantage of information and communication technologies to simplify the access to healthcare by patients. With the publication of Regulation (EU) 2025/327 on the European Health Data Space (EHDS), telemedicine has gained new momentum, particularly in the context of MyHealth@EU.

**Objectives:**

This article explores the framework for cross-border telemedicine under the EHDS, with a focus on real-world applications. It also aims to identify the enablers and barriers in several key domains, including legal, regulatory, organizational, financial, clinical, cultural, and technical aspects. The article also aims to discuss innovations in the field of telemedicine, namely the application of artificial intelligence.

**Methods:**

This review was conducted, focusing on cross-border telemedicine applications and the impact of the EHDS. The review also included studies related to telemedicine implementations in different medical disciplines, presenting the key successes and the challenges associated with these methods.

**Results:**

It is highlighted the initial progress made in cross-border telemedicine, where various approaches have been used, including teleconsultation, tele-expertise exchange, telemonitoring, telepathology, teleradiology, and remote surgery. Despite challenges such as legal uncertainties, financial constraints, and technical barriers, the integration of EHDS, supported by MyHealth@EU, has proven beneficial in building trust in secure, reliable telemedicine applications. The lessons learned and recommendations offer valuable insights for scaling cross-border telemedicine services. With the implementation of the EHDS and the use of MyHealth@EU, services can significantly improve access to healthcare and clinical outcomes by enabling more informed decision-making. As these services continue to evolve, they will contribute to a more integrated, and patient-centered healthcare system.

## 1 Introduction

In today's interconnected world, telehealth has emerged as a transformative trend, recognized as a potential game changer in healthcare (1). Its use has more than doubled across WHO European Regions (2). Telehealth refers to the remote delivery of healthcare services using information and communications technologies (ICT) and encompasses a wide range of activities, including clinical services and non-clinical services such as health education (for the public and professionals) and administrative meetings. Telemedicine, as a subset of telehealth, focuses specifically on the use of ICT for clinical care, including diagnosis, treatment, and prevention of diseases (3–5). These services can take various forms, such as provider-to-provider or provider-to-patient care, and can be delivered synchronously (e.g., live video consultations) or asynchronously (e.g., store-and-forward methods, where information is reviewed at a later stage) (6, 7).

The rise of telemedicine can be attributed to several factors, including advancements in digital technology, the global expansion of internet access, and the increasing demand for accessible healthcare services, especially due to pressure exerted by the COVID-19 pandemic (8). Over the past few decades, the integration of digital tools into healthcare has facilitated more efficient care delivery, particularly in underserved regions or areas with a shortage of medical professionals (4).

Cross-border applications of telemedicine have gained significant attention in recent years, particularly within the European Union (EU) and other regions with interconnected healthcare systems (9). This trend has been driven by several factors, such as limitations in healthcare resources and citizens mobility across countries. Recognizing its strategic relevance to healthcare, telemedicine was introduced in the European Health Data Space (EHDS) Regulation (EU) 2025/327 (10), in connection with the MyHealth@EU infrastructure. This regulation plays a central role in enabling the secure exchange of health data across EU countries, supporting both in-person and virtual care. This EU initiative is key to ensuring a continuous and coordinated healthcare provision, providing citizens with improved access to their own health data, and reducing fragmentation in healthcare systems. These advantages are framed within the relevant regulations that govern data privacy and security, in a complex landscape with varying levels of digital maturity among countries (9).

The purpose of this review is to provide an overview of the applicability of telemedicine within the framework of the EHDS Regulation. It examines existing cross-border studies in different medical specialties in which telemedicine was the primary focus. It also identifies key enablers and barriers that affect the adoption and deployment of telemedicine in cross-border contexts and offers recommendations for addressing these challenges to enhance the successful implementation of telemedicine services.

## 2 Methods

The studies were identified based on a period from June 2009 to June 2025 as it ensures a broad analysis of cross-border telemedicine during a period marked by significant technology, regulatory and policy evolutions. It also provides a comprehensive view of pre- and post-COVID-19 scenarios. The databases used were PubMed/MEDLINE, Science Direct, IEEE Xplore, and Google Scholar. To ensure comprehensive coverage, the following Boolean search strategy was used: (“telemedicine” OR “telehealth” OR “remote care”) AND (“cross-border”) AND (“teleconsultation” OR “tele-consultation” OR “telesurgery” OR “teleradiology” OR “telepathology”).

Studies retrieved through this search were screened for relevance based on their titles and abstracts. Full-text studies were then assessed for eligibility according to the inclusion and exclusion criteria described below. Duplicates and studies that did not meet the eligibility criteria were excluded using Zotero. The inclusion criteria focused on peer-reviewed case descriptions of telemedicine applications involving two or more countries, as well as European regulations and organizational publications regarding the applicability of telemedicine; while exclusion criteria targeted studies that did not focus on cross-border telemedicine applications, lacked clear methods for implementing telemedicine in real-world scenarios, were not peer-reviewed to a satisfactory standard, or were not aligned with the scope of EHDS Regulation. Additional databases, such as EUR-Lex, the Organization for Economic Co-operation and Development and the World Health Organization, were also consulted to identify relevant standards, guidelines, and legislation related to telemedicine. Because these platforms offer limited search functionalities and differ in scope, the searches were performed using broader and more adaptable terms: “telemedicine”, “telehealth”, “cross-border”. Additional studies were included to complement the initial review, particularly for the analysis of barriers, enablers, and emerging opportunities for cross-border telemedicine, such as artificial intelligence.

**Figure 1:**
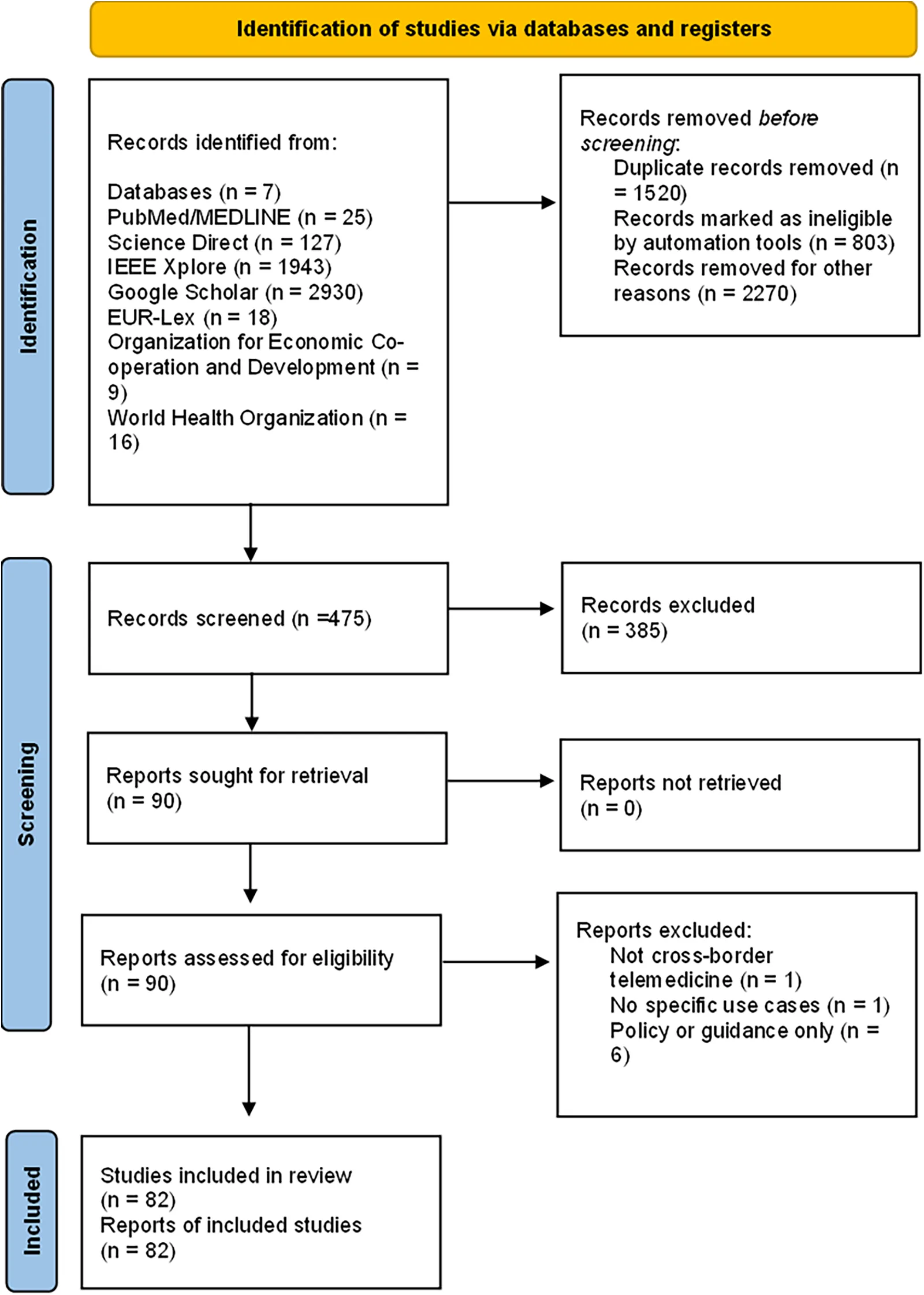
PRISMA2020 flow diagram of data collection and analysis, showing included reports/studies/use cases based on the inclusion and exclusion criteria listed in the methodology section.

A total of 3,843 studies were identified through the search. After removal of duplicates and screening for relevance, 90 studies were assessed for eligibility (Figure 1). Of these, 82 studies were included in the final review based on the criteria outlined above. Data were extracted from the included studies using a standardized form that captured details such as study design, population characteristics, telemedicine interventions, outcomes, and implementation strategies. The extracted data were then synthesized thematically to identify key barriers, enablers, and opportunities for cross-border telemedicine.

## 3 The transition to EHDS and the implementation of cross-border digital health services

MyHealth@EU infrastructure, formerly known as the eHealth Digital Service Infrastructure (eHDSI), was established under Article 6 of Directive 2011/24/EU (11), which addresses patients' rights in cross-border healthcare. Developed through joint efforts of EU Member States, this infrastructure enables patients to share their electronic health data with healthcare providers while traveling abroad. The implementation of National Contact Points for eHealth (NCPeH) in EU Member States was crucial for facilitating communications through MyHealth@EU, ensuring efficient, secure, and interoperable healthcare connectivity across participating countries. The first services included exchange of the electronic Patient Summary (PS) and Prescription and Dispensation (eP/eD); at the time of writing, the voluntarily participating countries were Cyprus, Czech Republic, Estonia, Spain, Finland, France, Greece, Croatia, Ireland, Lithuania, Luxembourg, Latvia, Malta, Netherlands, Poland, and Portugal.

Originally based on voluntary participation, MyHealth@EU will become mandatory for all Member States by 2029 under the EHDS Regulation (Articles 23 and 105), enabling better patient-centered healthcare through cross-border sharing of patients' health data when needed (10, 12, 13). Specifically, per Article 105 of the EHDS Regulation, the exchange of PS and eP/eD – classified as priority categories of personal electronic health data under Article 14 – is expected to be fully operational across all EU by 2029. This obligation primarily requires Member States to enable health data exchange, while electronic health record (EHR) system manufacturers must ensure conformance for systems processing these categories at the same deadline. In addition, other health data types – including medical test results, including laboratory and other diagnostic results and reports, discharge reports, and medical imaging studies and related imaging reports – are scheduled for implementation by 2031. These timelines are supported by implementing acts under key Articles (Articles 13, 15, 23, and 36), which specify technical and interoperability requirements.

The EHDS Regulation also enables Member States to offer supplementary services via MyHealth@EU. These include support for telemedicine, mobile health, citizens' access to translations of their health data, and exchange/ verification of health-related certificates, among others. Moreover, Recitals 28 and 29 highlight telemedicine's growing significance for improving healthcare access and reducing EU disparities.

While the EU has competence to regulate consumer protection and the internal market, healthcare policy remains largely under Member States' jurisdiction per Article 168 of the Treaty on the Functioning of the European Union (14). However, national policies must not impede free movement of health data, particularly in cross-border care where seamless electronic health data access safeguards service quality, including in telemedicine. For instance, a patient seeking a teleconsultation with a specialist in another Member State could enable access to their translated health data via MyHealth@EU.

Thus, the EHDS Regulation lays key foundations for EU health system interoperability, particularly via the European Electronic Health Record Exchange Format (EEHRxF) under Article 15 (10). Overall, it promotes public health, especially post-COVID-19 challenges best addressed at EU level due to their cross-border nature and the free movement of people, goods and services (14, 15). It also emphasizes data protection and safeguarding citizens' private information under the General Data Protection Regulation (GDPR) [Regulation (EU) 2016/679] (16). EHDS provisions safeguard citizens by ensuring that digital health data processing respects privacy (e.g., via anonymization/pseudonymization of data for secondary use) (10, 17). The EHDS Regulation also emphasizes the critical need for secure identification and authentication in remote cross-border contexts to ensure that only authorized individuals can access sensitive health data. The European Commission's role in adopting implementing acts to facilitate these processes is also highlighted, ensuring that both health professionals (HP) and patients can navigate the complexities of cross-border health data exchange with confidence and security.

Therefore, while the adoption of MyHealth@EU to support cross-border telemedicine is not mandatory, it is incentivized due to telemedicine's growing importance in enhancing healthcare accessibility and continuity. By enabling existing MyHealth@EU services to support cross-border telemedicine, Member States can leverage a trust-based framework that facilitates access to health information, upholds patient's rights to share their health data, and promotes quality of care for patients seeking telemedicine services abroad. This aligns with the EU's broader goals of ensuring free movement of citizens and the effective use of digital healthcare solutions. While this integration opens new opportunities for the healthcare sector, it also presents challenges that must overcome the potential of cross-border telemedicine.

This review aims to present practical examples of telemedicine services in cross-border context to highlight key enablers and identify barriers that must be overcome to fully leverage the telemedicine's potential and support the EHDS Regulation implementation. Based on the selected studies, the analysis below is organized into three key areas: i) real-world cross-border telemedicine pilots and studies, ii) main enablers and barriers to cross-border telemedicine, and iii) artificial intelligence (AI) as an opportunity to streamline telemedicine implementation.

## 4 Telemedicine as an emerging cross-border tool to promote better healthcare

The impact of telemedicine on improving access to healthcare and therapeutic outcomes can be examined from points of view and extended to cross-border contexts (18, 19). Telemedicine enables remote healthcare delivery, allowing individuals in remote or underserved regions to access services that might otherwise be unavailable. It is particularly beneficial for citizens with mobility limitations and for the continuous monitoring of patients with chronic diseases, regardless of their location. Moreover, it may reduce the need for HP to travel to hard-to-reach or rural areas, thereby contributing to cost reductions (20). Telemedicine tools can also support the exchange of medical expertise across borders, fostering collaboration among HP. Through these advantages, telemedicine has been shown to improve patient adherence to therapeutic plans and lead to better therapeutic outcomes by enhancing patient engagement and supporting more effective disease management (18, 21).

A compendial study by Zhang et al., presents a telemedicine success model that highlights the vital role of interoperability within her systems in enhancing the design and framework of telemedicine approaches, with the goal of improving both patient outcomes and clinician experience. The model identifies three key quality components that must be addressed: (i) health information, (ii) the EHR system, and (iii) telemedicine service. Ensuring interoperability across these areas enables seamless data exchange, supporting coordinated, value-based care and improved patient safety. Integrated EHR systems facilitate accurate information flow across care settings, which has been associated with reduced mortality and better quality of care for acute conditions. These findings are supported by core mechanics that enable the scalable telemedicine implementation and show how clear regulatory requirements and timetables can reduce the administrative workload associated with telemedicine services, help mitigate professional burnout by streamlining workflows, and encourage further investment in health information technology (IT) infrastructure. Furthermore, addressing these factors ensures that telemedicine interoperability expands access in underserved rural areas, improves mental health through better monitoring, and strengthens healthcare systems for elderly. Thus, telemedicine contributes to reducing healthcare disparities between rural and urban areas (22).

### 4.1 Real-world applications of cross-border telemedicine

Studies that report on the real-world applications of cross-border telemedicine are crucial for demonstrating its potential to support healthcare delivery worldwide. The selected studies are organized into three areas of cross-border telemedicine: (i) applications supporting telemonitoring and teleconsultation for disease management, (ii) applications in pathology and radiology, and (iii) applications in surgical procedures.

#### 4.1.1 Telemonitoring, teleconsultation and tele-expertise for disease management

For patients with chronic illnesses that require special care or frequent consultations with their HP, telemedicine can significantly improve safety monitoring and follow-up. In this context, health data can be transmitted digitally and continuously, allowing early identification of potential disease flare-ups (23).

The review by *Peyroteo* et al. analyzed Health Remote Monitoring Systems (HRMS) in primary health care for chronic disease management. HRMS use sensors, wearables, and Internet of Things (IoT) technologies to enable continuous remote monitoring and personalized care, with a primary focus on diabetes and cardiovascular diseases. Despite growing interest and adoption, the review highlights significant challenges in integrating HRMS into existing healthcare practices, workflows and infrastructures. A key limitation is the difficulty of aligning these technologies with the clinical processes and routines of multidisciplinary teams, which hinders scalability and widespread use. Additionally, the integration of HRMS-generated data into existing primary healthcare communication and information systems is often insufficient, resulting in fragmented data and reduced clinical value. These issues are further exacerbated in cross-border contexts, where ensuring interoperable data adds an additional layer of complexity. The study suggests that involving HP and patients in the design and implementation of HMRS is crucial for usability, clinical relevance, and adoption. While pilot studies demonstrate potential benefits, the review concludes that robust long-term evidence on the effectiveness, cost-efficiency, and broader impact of HRMS in primary health care management remains limited (24).

Similarly, a study by *Shaw* et al. described the Expanding Technology-Enabled Nurse-delivered Chronic Disease Care (EXTEND), a randomized telemedicine trial that integrates mobile monitoring devices into a nurse- and pharmacist-led care system for patients with poorly controlled diabetes and hypertension (25). Four devices were selected: a glucometer, blood pressure cuff, scale, and activity tracker, and configured to transmit patient data directly into the EHR via the Validic platform (26). Health professionals used an interactive EHR dashboard to monitor disease trends and receive alerts for values outside safe thresholds. Nurses conducted regular telemedicine encounters to review data and collaborate with pharmacists on medication management. By integrating mobile monitoring data into patients' EHR and leveraging the EXTEND randomized clinical trial, this model enhances both clinical relevance and scientific rigor. Integrating real-world patient data into clinical systems supports timely decision-making and enables more accurate, evidence-based interventions. Combined with its nurse- and pharmacist-led design applied to diverse populations, the model demonstrates scalability and promotes health equity. It exemplifies how telemedicine can enhance chronic disease management.

Another study by Mohr et al. evaluated the impact of home telemedicine monitoring on healthcare utilization and mortality among patients aged 65+ with congestive heart failure (CHF), chronic obstructive pulmonary disease (COPD), or diabetes mellitus (DM). The study compared two groups over 12 months: patients receiving dedicated monitoring (remote devices and nurse care coordination within healthcare institutions) vs. controls without telemonitoring platforms. Telemedicine initiation was associated with increased emergency department visits across all three conditions; hospital admissions remained unchanged for CHF and DM but showed a modest increase for COPD. Importantly, home telemedicine users with CHF and DM had significantly lower all-cause mortality, whereas COPD patients showed increased mortality. The study suggested that telemedicine may function effectively as an early warning system, improving survival in CHF and DM through timely interventions but showing less clear benefits in COPD management (27).

A study by Rodríguez-Valero et al. evaluated the effectiveness of the TRIP Doctor® smartphone app in providing telemedicine support to international travelers. The study monitored 449 adult travelers at Hospital Clinic Barcelona Travel Clinic, in Spain, using the app for daily health monitoring and remote medical advice via integrated chat with specialized physicians (teleconsultation). Of these, 13% used the chat system for telemedical assistance, primarily for mild symptoms such as diarrhea, skin conditions, and fever. Most cases (90%) were resolved remotely without local doctor referral, with symptomatic treatment prescribed in 38% of consultations. Only 10% required local doctor referral (primarily for fever or severe diarrhea), and 13.5% were referred back to the travel clinical upon return. Longer trip duration (>14 days) was associated with increased telemedicine use. These findings indicate that smartphone app-based telemedicine can effectively manage common travel-related ailments remotely, reducing the need for in-person care during travel (28).

A meta-analysis carried out by Janjua et al. evaluated the effectiveness of telehealth interventions, including remote monitoring and multi-component programs, for patients with COPD. The analysis included 29 randomized controlled trials with over 5,600 participants. Most interventions employed asynchronous remote monitoring combined with usual care or alone, while a minority used real-time data review. Overall evidence quality was low to moderate, indicating that remote monitoring added to usual care probably reduces COPD-related hospital readmissions but has little or no effect on exacerbation rates, health-related quality of life, dyspnea, overall hospital admissions, or mortality. Multi-component interventions may yield short-term quality of life improvements and reduced hospital re-admissions but have unclear long-term benefits. No harm or adverse effects were reported. The study highlights telehealth as a potentially useful adjunct, though larger studies are required to identify which patient subgroups may benefit most (29).

A study conducted by Khan et al. demonstrated the importance of international collaboration between low and high-resources settings in patient treatment. With the patient's consent and via teleconsultation with anonymized data exchange, a Nigerian clinician collaborated with physicians in England for patient care. The collaboration sought assistance in diagnosing patients with complex medical history. Following diagnosis and surgical intervention, the patient showed a positive response to the treatment. Such collaborative frameworks can model global health management by addressing increasing healthcare demands, optimizing time and cost efficiency by eliminating the need for patient and physician travel, and facilitating medical knowledge exchange (30).

Ramnath et al. described the implementation of a hybrid critical care monitoring program combining in-person support and Tele-ICU services across three United States of America (USA)-Mexico border hospitals during the COVID-19 pandemic, with a focus on assessing the impact of cross-border monitoring. The intervention included direct telemedicine management at the USA hospital and consultative, case-based tele-education in the two Mexican hospitals, primarily due to licensure constraints. Findings indicated improved adherence to evidence-based critical care practices and increased staff confidence in managing COVID-19 patients through enhanced monitoring and follow-up without increased workload. The hybrid approach fostered trust, enabled bidirectional knowledge exchange, and addressed resource and cultural disparities (31).

Other comparable studies have similarly demonstrated the feasibility and impact of cross-border telemedicine in resource-constrained settings. Bustamante et al. analyzed USA-Mexico healthcare interactions post-COVID-19, highlighting telemedicine strategies to improve access for Mexican migrants returning from the USA, and emphasizing regulatory flexibility and telemedicine's role in strengthening primary care pathways across borders (32). Likewise, Mishori et al. evaluated mental health assessments conducted remotely by USA clinicians with Mexican asylum seekers in detention or custody, a model described as “better than having no evaluation done”. Their findings showed that remote assessments largely achieved diagnostic goals, helped establish rapport, and offered a practical solution for reaching hard-to-serve cross-border populations (33). Together, these studies reinforce how telemedicine can bridge regulatory and geographic barriers, support clinical decision making, and facilitate knowledge exchange in underserved cross-border healthcare monitoring (33).

A comparative observational study conducted by Rodríguez-Ortega et al., between 2013 and 2022, analyzed usage trends of a telemedicine tool for clinical advice among Cameroonian HP, particularly nurses, participating in the “Telemedicine: health that connects” program in sub-Saharan Africa. The program enables users to request advice from volunteer Spanish medical specialists via the “diagnosis assistance” (DA) telemedicine tool. The study included 296 health professionals (77 nurses; 59% women) and found nurses accounted for 68% of the 2,527 DA requests. Nurse-driven DA requests showed an increasing trend in internal medicine, obstetrics, gynecology, and dermatology. The platform was valued for diagnostic, preventive, and training benefits, though major limitations included internet costs (38%), quality (53%), and time constraints (77%). Health professionals reported reduced hospital referrals and patient recovery times. Overall, the study demonstrated telemedicine's feasibility and effectiveness for improving clinical decision-making among rural Cameroonian nurses in resource-limited settings. The authors emphasized that long-term success requires healthcare centers and governments to prioritize adoption, support infrastructure, and uphold ethical data privacy standards. Notably, 62% of participants reported high center leadership commitment, crucial for program sustainability (34).

The study by Troschke et al. examined the creation of a German–Polish telemedicine network for pediatric oncology and hematology in Euroregion Pomerania. The network sought to improve patient care through cross-border medical case reviews, family involvement, and HP education. The Temicare project, funded by Interreg-VA program, connects institutions in Greifswald, Szczecin, and Kraków, for real-time consultations and medical image sharing. It provided hybrid seminars and case presentations for physicians, nurses, and students, focusing on topics like immunotherapy. English served as the primary language for case discussions and partner meetings, while Polish and German were used for family meetings with interpreter support. Results showed successful integration, with 90 events held and 2,295 participants. Pre- and post-seminar tests demonstrated knowledge improvements. Families reported greater confidence in their children's treatment. Despite challenges such as high coordinative and time demands, data privacy concerns, and differences in work/training structures, the project demonstrated telemedicine's effectiveness in enhancing cross-border pediatric care and offers a model for other regions. Key enablers included financial funding, translator availability, baseline English proficiency, and effective interpersonal exchange (35).

#### 4.1.2 Telemedicine applied to radiology and pathology

The increasing demand for diagnostic services in remote and underserved regions, coupled with the global shortage of specialists, has catalyzed telemedicine evolution. In this context, teleradiology and telepathology emerge as pivotal domains, enabling transmission and interpretation of diagnostic data across geographical boundaries (36).

Several teleradiology studies highlight the benefits and challenges of this telemedicine domain, such as the Eurasian teleradiology program, a collaborative initiative across Central Eastern Europe and Asia designed to support medical centers in interpreting high-resolution computed tomography (HRCT) scans for the presence of usual interstitial pneumonia in suspected idiopathic pulmonary fibrosis (IPF) cases. Weikert et al. analysed data collected from January 2014 to May 2019, involving HRCT from 239 medical centers across 12 European and Asian countries. Images were securely transmitted through the Picture Archiving and Communication Systems (PACS) for expert review, using a structured report template developed by a board-certified radiologist (>20 years' cardiothoracic imaging experience) and an interstitial lung disease pulmonologist. Among 703 patients' HRCT data, image transmission errors occurred in only 7.1% of cases, and were resolved through referring site-IT collaboration, demonstrating network reliability. The program achieved its 5-business-day reporting target in 88.2% of the cases, with an average turnaround time of 1.7 business days. A survey of referring physicians revealed high satisfaction rates with the centralized expert teleradiology program, with many noting its positive impact on enhancing local IPF diagnostic expertise. Reports were generated in English to mitigate language barriers between centers, further facilitated by a structured report template ensuring consistency and clarity. Survey results indicated that trust is established through high-quality reports and dependable turnaround times, fostering referring physicians' confidence. The authors highlighted additional success factors for the cross-border teleradiology program: clearly defined responsibilities (including one program coordinator), efficient communication channels, and secure image transmission ensuring data privacy. Overall, leveraging these elements enabled timely and accurate diagnoses, enhancing global patient care and management (37).

Ross et al. evaluated the Baltic eHealth and R-Bay projects, which enabled remote interpretation of knee and hip x-rays between partner countries. These represented early explorations of cross-border telemedicine feasibility but were limited in scope, both clinically and technically. Clinical partners from multiple countries reviewed cases to build trust and standardize reporting. The technical setup included secure data transfer, streaming, workflow integration, and a structured reporting tool to address language barriers, the primary challenge. Although successful, the service was limited to knee and hip x-rays due to reporting tool constraints. Legal, security, and financial aspects varied across countries and were not fully addressed, though EU-level efforts to harmonize cross-border eHealth have since begun (38).

Parkes et al. examined telemedicine interventions in six conflict-affected WHO Eastern Mediterranean Region (EMR) countries: Afghanistan, Gaza, Iraq, Libya, Syria, and Yemen. The study highlighted telemedicine applications in radiology, dermatology, mental health, and intensive care, primarily facilitated by USA and European humanitarian/academic organizations to bridge physician access gaps. The goal of the interventions was to bridge gaps that existed due to the inaccessibility of physicians in the region. Key challenges included limited internet bandwidth, unreliable electricity, and shortages of trained HP. Most interventions used cost-effective technologies like email and social media platforms, compatible with mobile devices. Barriers included insufficient funding, volunteer reliance, and the lack of evaluation mechanisms, impacting sustainability of these projects. Patient outcomes were reported, but few studies assessed the long-term impact or ethical concerns like data privacy. The review concluded that although telemedicine can provide vital healthcare support in conflict settings, more comprehensive evaluations, better governance, and sustainable models are needed to ensure effectiveness and longevity. Further research should focus on localizing telemedicine strategies and addressing gaps in ethical standards and data security (39).

In terms of quality assurance (QA), an article by Hetenyi et al. highlighted the development and rigorous implementation of a sub-specialized and cross-border teleradiology service at the Telemedicine Clinic (TMC), one of Europe's largest teleradiology providers. TMC offers high-quality radiologic reporting through collaboration between radiologists and healthcare institutions in Europe and Australia. Daytime elective reporting is done from Europe, while night-time emergency services are covered by European-accredited radiologists based in Australia, enabling continuous 24-hour coverage. A key element is TMC's custom-built radiology information system called Optemis, which automates case distribution and supports peer review. The QA framework encompasses rigorous radiologist selection, structured reporting, continuous feedback, and second readings, leading to a low clinically relevant disagreement rate of 4%. This international collaboration model exemplifies how sub-specialization and quality assurance drive safe and efficient cross-border telemedicine (40).

On the domain of privacy and security in teleradiology, Pekka Ruotsalainen defined key requirements for a trusted implementation, which include a harmonized security policy adopted by all entities, adherence to core privacy principles (e.g., fair processing, purpose limitation), and formalized contracts defining responsibilities between data controllers (e.g., hospitals) and processors (e.g., radiologists). Technical safeguards, such as strong authentication, end-to-end-encryption and audit logs, must be risk-based and legally compliant. Cross-border operations require contractual guarantees of equivalent data protection, prohibition of secondary data use, and anonymization/pseudonymization where feasible. System-wide certification is critical for trust verification (41).

These services not only optimize healthcare delivery but also present complex technical, legal, and ethical challenges. The European Society of Radiology White Paper exemplifies the importance of regulation in this area, establishing foundational governance principles for cross-border teleradiology, including standardized reporting protocols, adherence to international guidelines (e.g., GDPR, ISO), and formal accreditation systems to ensure quality and interoperability across jurisdictions (42).

Recent advances in digital health infrastructure have facilitated the development of sophisticated telepathology platforms. These systems increasingly incorporate diagnostic image viewers, cloud-based storage solutions, open-access interfaces, modular plug-in functionalities, and mobile-enabled devices. Through such platforms, referring medical centers can digitize and transmit histopathological slides to specialized pathology departments for remote evaluation and reporting, significantly enhancing diagnostic timeliness and workflow efficiency (43, 44).

The *Patologi Oltre Frontiera* initiative, led by Italian pathologists, established a virtual laboratory dedicated to the remote analysis of stained histological specimens. This program aimed to address pathology service shortages in under-resourced regions by providing remote diagnostic support to local clinicians. A notable implementation occurred in Zambia, where surgical specimens were prepared and digitized using whole-slide imaging technology. These digital slides were transmitted via satellite connection to Italy, where two independent pathologists performed comprehensive remote evaluations. The resulting diagnoses were relayed back to the Zambian medical team through secure internet channels, enabling timely clinical decision-making despite lacking on-site pathology services. Building on this foundation, Pagni et al. (using data from April to October 2007) compared telepathology diagnoses with traditional microscopy in this program. The results showed a high correlation between the two methods, with no discordance in cytologic specimens. However, descriptive differences showed telepathology-to-microscopic review discordance rate of around 12.3%. Key constraints included initial infrastructure setup costs at local hospitals, especially in under-resourced regions, plus ongoing maintenance and communication costs (43).

Having these examples in mind, it is clear that one of the principal advantages of telepathology lies in its capacity to enhance operational efficiency across diagnostic services. By eliminating the need to physically transport fragile glass slides via third-party courier services, a process often characterized by high costs, logistical complexity, and time delays, telepathology significantly streamlines diagnostic workflows. For healthcare institutions lacking in-house subspecialty expertise, remote pathology consultation is a critical enabler of timely and accurate diagnosis. Such collaborative arrangements have demonstrated utility in low- and middle-income countries, where they facilitate the exchange of laboratory best practices, promote continuing medical education, and support collaborative research initiatives. In some cases, telepathology has been integrated into broader telemedicine frameworks, functioning alongside disciplines such as teledermatology to create comprehensive, institution-wide digital health ecosystems (43, 44).

#### 4.1.3 Telemedicine applied to surgical procedures

Telesurgery is the act of performing surgery remotely, through the utilization of a robotic interface and setup in two different locations that allows complete remote control by HP. This is an emerging technology whose applicability is growing due to improvements in communication infrastructure response times, which, with current fiber optic networks and 5G communication, allow for limitless cross-border collaboration and specialization of robotic tools. One benefit of this technique is that, because operation involves a robotic interface, surgeons can practice indefinitely in virtual reality setups before performing the actual inpatient surgery (45, 46).

In 2001, a compendial study by Marescaux *et al*. demonstrated the feasibility of the first remote robot-assisted laparoscopic cholecystectomy using a high-speed terrestrial optical-fiber network that transported data through dedicated connections using asynchronous transfer mode telecommunication technology. This groundbreaking procedure was performed between two cities, New York and Strasbourg, on a porcine model. The surgery was completed in 54 min with a mean transmission delay of 155 milliseconds (ms), ensuring real-time surgical control. The robotic system, comprising a surgeon console in New York and a patient-side setup in Strasbourg, allowed easy manipulation of instruments without difficulties or missed inputs. This approach was later successfully applied to a 68-year-old female patient, who underwent remote laparoscopic cholecystectomy with surgeons in New York and the patient in Strasbourg. The procedure used a 16-minute circuit delay, with gallbladder dissection completed in 54 min and no intra-operative complications. The patient's post-operative recovery was uneventful and without complications (47).

Aldousari et al. demonstrated the feasibility of cross-border telesurgery with a robotic-assisted radical prostatectomy performed between Kuwait and Shanghai, using low-latency connectivity via fiber optic broadband and 5G backup to transmit surgeons' inputs to the robotic probe. The 2024 procedure involved a surgeon in Shanghai operating remotely on a patient in Kuwait, with an average round-trip latency of 181.4 ms, ensuring real-time synchronization. The surgery was without complications, and the patient was discharged on postoperative day 2, with undetectable prostate specific antigen levels at seven weeks (48).

Telesurgery can also be combined with telemonitoring, as shown by a study published by Doering et al. The case study explored the factors facilitating the implementation of cross-border intra-operative teleneuromonitoring during aortic surgery, involving collaboration among hospitals from Germany, the Netherlands, and Switzerland. In this collaboration, thoracoabdominal aortic aneurysm repair is performed in Aachen, Hamburg, or Bern, while a neurophysiologist in Maastricht remotely monitors the patient's spinal function. This helps reduce the risk of paraplegia and paraparesis, common complications resulting from inadequate arterial supply. As this specialized surgery is rare and requires high expertise, it is not cost-effective to have a neurophysiologist in every surgical center. Instead, Maastricht's team, a leader in the field, provides remote monitoring to several European hospitals. Through semi-structured interviews with key actors, the study identified key factors for successful and sustainable collaboration. These included a clearly defined need that provided tangible benefits, such as cost-effective management of scarce specialized resources, and the establishment of agreed protocols and procedures to address healthcare system differences from the outset. This groundwork facilitated smooth collaboration despite national health system variations. Moreover, collaboration was driven by shared commitment to improving care quality and fostering innovation, sustaining long-term engagement. The importance of professional trust and recognition was emphasized, with collaboration initiated bottom-up by HP who trusted each other's expertise, highlighting mutual trust as essential for sustainable collaboration. While the study found the EU regulatory environment did not significantly impact collaboration, a call for clearer standardization and certifications, particularly for neuromonitoring training, was made. Key barriers identified included discrepancies in medical training and certification standards and the need for harmonized cross-border procedures. Overall, the study showed that willingness to collaborate, transparent communication, and cultivating trust were essential to overcoming barriers and ensuring initiative success (49).

 Table 1 synthesizes the analysis of real-world cross-border telemedicine applications, summarizing studies by geographical area, scope, and applicability. Additionally, the enablers and barriers identified in these telemedicine studies above serve as a starting point for the following section on the main enablers and barriers in implementing cross-border telemedicine services.

**Table 1:** Summary of the cross-border telemedicine use cases and their applicability in healthcare.

Author	Geographic coverage	Scope & applicability	Identified key enablers and barriers
Teleconsultation, tele-expertise and telemonitoring			
Rodriguez-Valero et al. (28)	Africa, Americas, Asia, Europe and Middle East	Scope: Telemonitoring and teleconsultation of travelers, allowing synchronous immediate remote assistance, referral to local doctors and follow-up post-return. Clinical Use: multiple diseases. Modality: Synchronous via smartphone app and chat.	Enablers: • Early clinical intervention • Reduction of unnecessary in-person consultations through adequate remote management • Enhanced identification and referral of patients requiring in-person evaluation • Remote prescription and management of treatment • Post-travel follow-up and continuity of care Barriers: • Uncertainty regarding scalability to other healthcare systems • Language barriers affecting referral processes • Limited applicability for international travelers • Lack of clarity on required cross-border agreements • Lack of information regarding interoperability requirements
Khan et al. (30)	Africa (Nigeria) and Europe (UK)	Scope: International collaboration and expertise exchange for diagnosis and treatment identification, and patient follow-up. Clinical use: Oncology. Modality: Asynchronous and synchronous.	Enablers: • Patient consent management • Collaboration between low- and high-resources settings enabling cross-border sharing of medical expertise, including multidisciplinary collaboration • Exchange of anonymized data • Reduced need for patient and HP mobility in complex medical cases, offering cost savings • Enhanced support for diagnosing and managing complex diseases Barriers: • Differences in patient consent management may hinder scalability due to legal and regulatory differences across countries • Requirement for formal cross-border agreements, potentially limiting expansion • Need for interoperable systems that enable secure health data exchange while ensuring data protection • Limited resources within pressured healthcare systems may constrain scalability • Lack of information regarding interoperability requirements
Ramnath et al. (31)	Mexico and USA	Scope: Tele-ICU intervention for cross-border patient mobility and expertise exchange/training. Clinical use: COVID-19. Modality: Asynchronous receiving patient data and direct care management.	Enablers: • Hybrid models that combine continuous monitoring, in-person support, and tele-ICU services • Enhanced cooperation in cross-border regions for disease surveillance and coordinated response • Implementation of evidence-based critical care practices, leading to increased staff confidence in patient management • Potential for maintaining or reducing workload through optimized resource allocation • Greater trust, bidirectional knowledge exchange, and efforts to address resource inequalities and cultural disparities Barriers: • Licensure constraints that limit the scope of practice and restrict scalability • Technological and infrastructure challenges affecting smooth implementation of telemedicine • Cultural and language differences requiring interpreters specialized in clinical terminology • Lack of information regarding interoperability requirements
Bustamante (32)	Mexico and USA	Scope: Cross-border patient mobility monitoring. Clinical use: COVID-19 (post-complications). Modality: Not specified.	Enablers: • Support in bridging regulatory and geographic barriers • Exchange of expertise facilitating clinical decision-making, and knowledge sharing in underserved, cross-border healthcare settings • Policy reforms for more flexible migrant-tailored health insurance enrolment allowing dynamic inclusion during cross-border movement Barriers: • Complex enrolment procedures and regulatory gaps for transnational patients, resulting in a lack of coherent regulatory framework for cross-border care • Lack of information regarding interoperability requirements
Mishori et al. (33)	Mexico and USA	Scope: Cross-border patient mobility mental health evaluation and support. Clinical use: Mental health. Modality: Synchronous video evaluations.	Enablers: • Remote evaluations enabled through coordinated efforts among clinicians and legal teams • Reduction in travel by permitting convenient off-site assessments • Access to expert clinicians through remote evaluation Barriers: • Variability in legal admissibility depending on jurisdiction, given uncertainty regarding long-term equivalency of remote vs. in-person affidavit • Limitations in physical examination and visual assessment, including difficulty with assessing body language and motor activity, especially with poor or inadequate video quality
Rodríguez-Ortega et al. (34)	Africa (Cameroon) and Europe (Spain)	Scope: International collaboration and expertise exchange for patient care. Clinical use: Multiple diseases. Modality: Asynchronous via digital platforms.	Enablers: • Exchange of expertise between high- and low-resource countries • Reduction of hospital referrals and patient recovery times • Organizational endorsement and support from HP • Positive user acceptance due to practical benefits in diagnosis, treatment and training Barriers: • Limited broader access due to costs related to internet access and device procurement • Limited digital literacy impeding optimal platform use and networking • Restricted patient engagement due to time constraints linked to clinical workload • Limited scalability due to the usage of a specific platform • Adoption and sustainability dependent on prioritization by centers and government, infrastructure availability, as well as ethical and data privacy standards
Troschke et al. (35)	Europe (Germany and Poland)	Scope: International collaboration and expertise exchange for diagnosis and treatment. Clinical use: Pediatric oncology and hematology. Modality: Not specified.	Enablers: • Border countries cooperation • Funding availability • Cross-border medical case reviews, family involvement and education for HP • Clear definition and adequate knowledge of a common working language • Use of interpreters as communication bridges between patients and HP Barriers: • High coordination and time demand • Concerns about data privacy issues • Differences in work and training structures • Possible limitations in scaling the platform to other regions • Fragmented health systems and lack of unified telemedicine protocols, contributing to interoperability challenges • Limited ability to perform comprehensive physical examinations • Incomplete or inaccurate patient data impacting the clinical outcomes • Relying on specialized interpreters leads to increased costs
Teleradiology			
Weikert et al. (37)	Central Eastern Europe and Asia	Scope: Radiologic analysis to support diagnosis. Clinical use: Respiratory diseases. Modality: Asynchronous image sharing.	Enablers: • Standardized report template in a common working language (e.g., English) that minimizes language barriers between centers and promotes clear, consistent, high-quality reporting across centers • Clear definition of responsibilities across the teleradiology network ensuring accountability and prompt responses • Errors promptly resolved through collaboration between the referring sites and information technology (IT) teams • Robust communication channels facilitating rapid interaction between radiologists and clinicians • Technology infrastructure supporting secure image sharing and remote access • Formalized workflows and quality assurance mechanisms ensuring reliable and trustworthy service, through defined procedure and timelines Barriers: • Logistical complexity in coordinating multinational multidisciplinary teams • Sustainability of the service is impacted by the limitation of funds
Ross et al. (38)	Europe (Czech Republic, Denmark, Estonia, Finland, Lithuania and the Netherlands)	Scope: Knee and hip x-ray analysis to support diagnosis. Clinical use: Orthopedic diseases. Modality: Asynchronous image sharing.	Enablers: • Technical setup to ensure secure data transfer, streaming workflow integration, and a structured reporting tool designed to overcome language barriers • Clear focus on maintaining clinical quality and building trust across international networks Barriers: • Limited in scope, both clinically and technically • Legal, security, and financial issues varying across countries, complicating broad service adoption
Hetenyi et al. (40)	Europe (Denmark, Spain Sweden, UK), Australia	Scope: Radiologic analysis to support diagnosis. Clinical use: Multiple diseases. Modality: Asynchronous image sharing.	Enablers: • Structured multi-level quality assurance (QA) system ensuring report accuracy and clinical relevance • Radiologist specialization, allowing focused expertise in specific imaging domains, with formalized training and qualification criteria • QA framework including rigorous radiologist selection, structured reporting and continuous feedback • Continuous radiology reporting ensured a 24/7 service by using European teams for daytime elective cases and Australian teams for night-time emergency services • Peer review and feedback ensuring service responsiveness and client satisfaction Barriers: • Complexity of aligning QA processes across multiple countries with differing standards • Potential resistance to feedback and peer review in a remote work environment • Lack of resource allocation for sustaining high QA standards in a commercial telemedicine environment
Telepathology			
Pagni et al. (43)	Africa (Zambia) and Europe (Italy)	Scope: Cytopathology and histopathology to support diagnosis and treatment. Clinical use: Multiple diseases. Modality: Asynchronous image sharing.	Enablers: • Use of virtual microscopy platforms enabling high-quality image digitalization and remote diagnosis • International collaboration providing specialized diagnostic support and mentoring to local pathologists, especially supporting pathology services in under-resourced regions • • Secure internet channels, thereby enabling timely clinical decision-making despite the limited/absence of on-site pathology services Barriers: • Limited local infrastructure including internet bandwidth and reliable electricity supply, especially in low-resource countries • Logistical challenges in specimen handling and slide digitalization under resource constraints • Financial sustainability concerns beyond the initial project funding • Regulatory and legal uncertainties related to remote diagnosis and data privacy in cross-border telepathology • Potential cultural and communication barriers between international experts and local health care providers
Telemedicine applied to surgical procedures			
Marescaux et al. (47)	USA and Europe (France)	Scope: Transatlantic telesurgery, robotic and computer technology. Clinical use: Gallbladder removal. Modality: Asynchronous image sharing.	Enablers: • Advanced robotic surgical systems capable of precise remote manipulation, allowing experts to deploy specialized skills remotely across borders without the need of travelling • Use of high-speed terrestrial optical-fiber network that transported data through dedicated connections using asynchronous transfer mode telecommunication technology • Rigorous pre-operative planning and synchronization between remote teams • Real-time surgical control Barriers: • High costs associated with robotic surgical systems and telecommunication infrastructure • Limited availability of suitable telecommunication infrastructure in remote or resourced-limited healthcare settings • Technical complexity demanding continuous maintenance, support and upgrades
Aldousari et al. (48)	Asia (China) and Middle East (Kuwait)	Scope: Prostatectomy, remote surgery, robotic surgery. Clinical use: Oncology. Modality: Synchronous image sharing for robotic surgery.	Enablers: • Low-latency, high-bandwidth fiber optic broadband networks with reliable 5G backup ensuring real-time responsiveness/synchronization • Careful pre-operative planning and intra-operative coordination between remote teams Barriers: • Requirement for highly reliable, low-latency telecommunication infrastructure, not available in all regions • Expensive robotic systems and network infrastructure requiring ongoing maintenance, support and upgrades • Complex training requiring effective operation and coordination of remote surgical procedures
Doering et al. (49)	Europe (Netherlands, Germany and Switzerland).	Scope: Teleneuromonitoring to support vascular surgery. Clinical use: Cardiovascular diseases. Modality: Synchronous image sharing for intra-operative teleneuromonitoring.	Enablers: • Cost-efficiency in managing scarce specialized medical professionals (e.g., neurophysiologist) enabling support in multiple centers, by leveraging existing resources from other centers • Establishment of agreed-upon protocols and procedures to address differences in healthcare systems from the outset • Shared commitment to improving care quality and fostering innovation, which helped to sustain long-term engagement among participants • Mutual professional trust and recognition to ensure sustainable collaboration, with the collaboration being initiated from the bottom-up by HP who trusted each other's expertise, highlighting that mutual trust is essential for a sustainable collaboration Barriers: • Limited impact of EU regulatory environment requiring clearer standardization and certifications • Need for fully interoperable health information systems that hospitals may still lack, especially in case of under-resourced regions • Discrepancies in medical training and certification standards, and the need for harmonization of procedures across borders
Combined telemedicine areas			
Parkes et al. (39)	Middle East and North Africa (Afghanistan, Gaza, Iraq, Libya, Syria and Yemen), Europe and USA	Scope: International collaboration and expertise exchange for patient care, especially in conflict areas (uses tele-expertise, teleconsultation, telemonitoring, telepathology and teleradiology). Clinical use: Multiple diseases. Modality: Asynchronous and synchronous depending on the needs.	Enablers: • Support for conflict-affected countries through cooperation • Telemedicine use for different medical specialties, enabling scalability and comprehensive utilization • Enabling healthcare delivery in conflict areas with limited physician access, and allowing flexible approaches for image exchange and video consultation, depending on available resources Barriers: • Insufficient infrastructure including poor internet connectivity, limited technology, and reliable electricity • Use of cost-effective technologies like email and social media platforms due to their accessibility and mobile compatibility, however limitations remain regarding confidentiality, ethical standards, and data security • Insufficient funding, reliance on volunteers, and the lack of evaluation mechanisms undermining sustainability • Lack of research on long-term impacts and ethical concerns such as data privacy • Lack of comprehensive evaluation, and need for a stronger governance, and sustainable models to secure effectiveness and longevity

Teleconsultation: A form of remote medical consultation in which HP use ICT to provide clinical advice, diagnosis, or treatment to patients or other health providers at a distance. This can include real-time (synchronous) consultations using video or audio links or store-and-forward (asynchronous) methods, where medical data, such as images or patient records, are shared for later review and response (4, 50, 51); Telemonitoring: Refers to the use of ICT by HP to monitor patients remotely. It involves continuous or regular collection of biomedical parameters, transmission and evaluation of health data to monitor the patient's state remotely. In telemonitoring, the data collection can be automatic or patient-guided (4, 50); Tele-expertise: A form of telemedicine in which a HP seeks a second opinion or expert guidance from a specialist remotely, typically using digital ICT. It allows for consultation across various clinical specialties’, enabling health providers to make more informed decisions without requiring the patient or specialist to be physically present (4); Teleradiology: Refers to the remote transmission of radiological images and patient data for interpretation, consultation, or review by a radiologist at a different location. It enables image reading across sites, often involving patient-identifiable information and cross-border data sharing (42); Telepathology: A form of interaction between HP involving the transmission of pathology digital images and related clinical information for different clinical purposes (52).

### 4.2 Main enablers and barriers in implementing cross-border telemedicine

**Figure 2:**
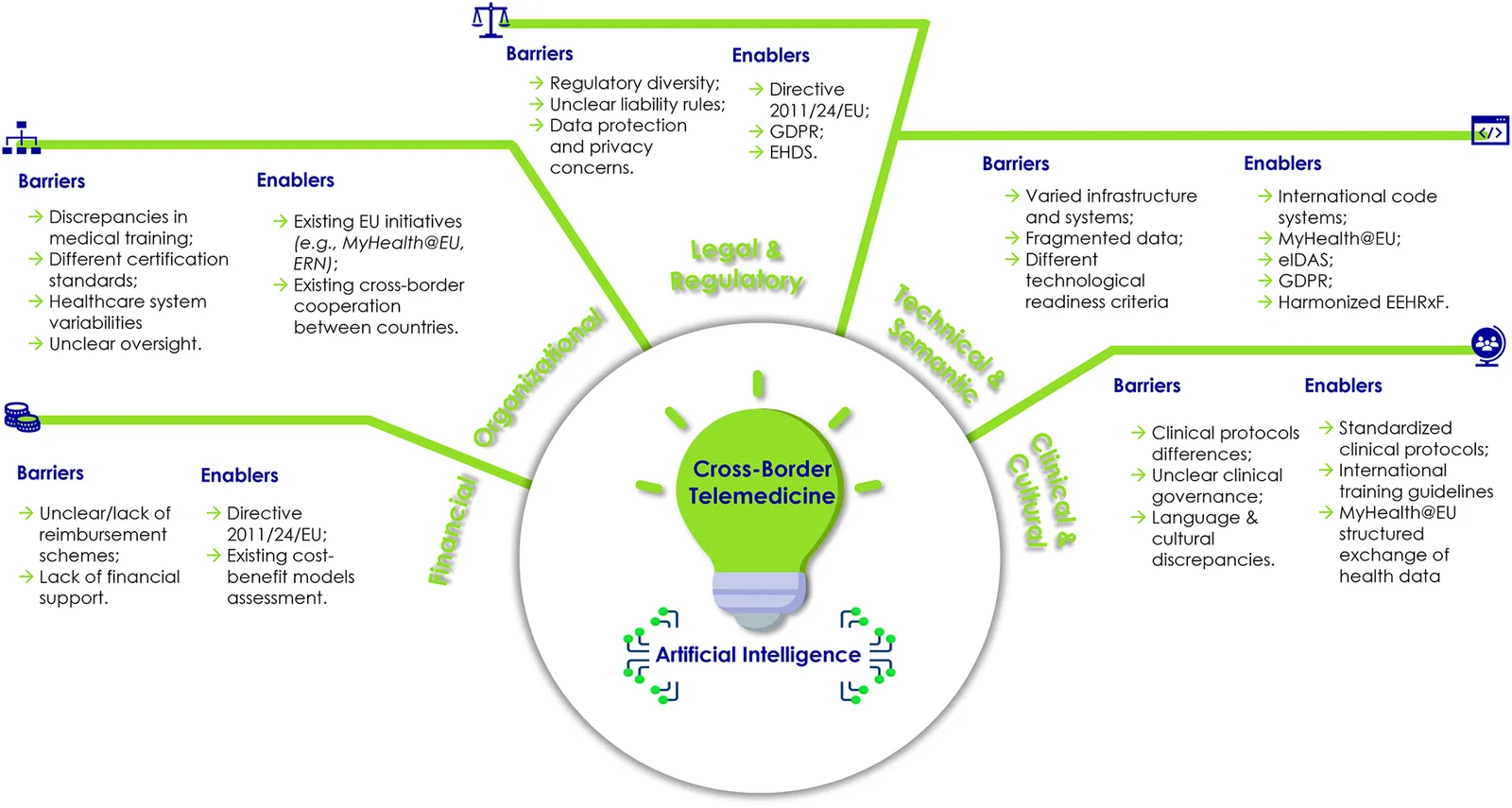
Overview of the main barriers and enablers associated with the implementation of cross-border telemedicine, divided in five compendial areas.

In order to successfully implement cross-border telemedicine services worldwide, it is important to recognize the main challenges associated with scaling-up these services. Addressing these challenges will be key to unlock the full potential of cross-border telemedicine in the long-term. This will empower healthcare systems, professionals and citizens with better and safer services, while also benefiting public authorities, insurers, and other stakeholders. Ultimately, this can foster more integrated and efficient healthcare services, particularly with the increasing burden on healthcare and the limited resources available worldwide and ensuring a smooth transition to a new era of health services (9, 53). These challenges must be addressed considering different layers. Figure 2 shows an overview of both enablers and barriers organized in five main layers, which have been analyzed to support the implementation of cross-border telemedicine services, with particularly focus on exploring critical elements to support the implementation of the EHDS Regulation. In addition, artificial intelligence (AI) was also analyzed as an opportunity to further support cross-border telemedicine.

#### 4.2.1 Legal & regulatory

Legal and regulatory challenges primarily stem from varying data protection and privacy laws across EU Member States and globally (16, 54, 55). This regulatory diversity and inconsistent harmonization create significant barriers to effective data governance, complicating health data exchange for cross-border telemedicine. These inconsistencies create diverse blockers that systematically hinder innovation in the field. Particularly, differing legal requirements create barriers for health professionals and patients, hindering seamless health data flow (56). Furthermore, aligning telemedicine with GDPR and relevant regulations while ensuring ethical and legal compliance remains complex despite EU-wide initiatives addressing new features in this area. Telemedicine as a supplementary MyHealth@EU service advances secure EU health data exchange, but challenges persist beyond technological capabilities.

Addressing these requires a robust legal and regulatory framework for cross-border telemedicine. This must provide clarity on legal, ethical, and data protection issues, with transparency on liability and jurisdiction. Clear malpractice rules with defined resolution and monitoring procedures, including audits, are essential. It should also guide cross-border telemedicine provision, promoting international collaboration (57). Legal safeguards include mutual licensure recognition per Directive 2005/36/EC on professional qualifications, since professionals may serve multiple countries (37, 40, 53, 57, 58). This requires local recognition and prerequisites such as language certificates to ensure patient-provider communication and care quality.

Transparent, well-defined informed consent is essential to ensure patients understand data use/sharing (53). Auditing must monitor health data use for accountability and compliance. The framework should outline data security, breach handling, and non-compliance penalties. This creates a secure, ethical, legally compliant environment protecting HP and patients.

While Directive 2011/24/EU, GDPR, and EHDS Regulation enable cross-border telemedicine, specialized frameworks must address telemedicine-specific requirements. These should include continuous monitoring for cybersecurity, ethical issues, and technological advancements.

#### 4.2.2 Organizational

The organizational layer plays a critical role in the successful implementation of cross-border telemedicine services, involving alignment of business processes, responsibilities, and expectations across healthcare entities to achieve mutually beneficial goals, as well as documentation and exchange of relevant information to enable smooth collaboration between HP, public administrations, and stakeholders in cross-border telemedicine services (34, 59). As demonstrated notably by Doering et al., well-defined need and tangible benefits were crucial first steps for successful collaboration among healthcare centers in the three involved countries (49). Establishing agreed-upon protocols and procedures was another key enabler cited across several studies to address healthcare system differences, such as varying medical standards or administrative practices, enabling smooth cross-border collaboration (37, 40, 49). This alignment facilitates efficient service delivery even amid differing healthcare systems across countries. Shared goals promote long-term organizational commitment, such as improving care quality, service range, and innovation. This commitment, alongside professional trust and recognition—including mutual recognition of certifications—supports sustainable collaboration. However, these examples involved limited institutions, highlighting the need for scalable procedures for broader implementation.

Discrepancies in medical training and certification standards create significant barriers, leading to inconsistencies in care delivery and complicating inter-organizational coordination. Variations in healthcare system structures further complicate coordination. Lack of centralized oversight and resistance to change from standardized procedures—particularly in regions prioritizing traditional face-to-face care—hinder implementation of cross-border telemedicine programs (37, 39, 40, 42). Overcoming these requires clear governance, harmonized training standards, and development of protocols and quality measures. Effective communication channels are crucial to facilitate cooperation across institutions and countries.

Integrating telemedicine into existing healthcare infrastructures, like MyHealth@EU, relies on well-defined organizational procedures agreed upon by Member States. Analyzing existing initiatives such as the European Reference Networks (ERN) and mobility mechanisms in the EU, including those for planned medical treatment abroad, helps identify best practices for cross-border care design and implementation (60, 61). These initiatives can be supported by cross-border telemedicine to facilitate seamless, efficient cross-border healthcare delivery.

#### 4.2.3 Financial

Most EU countries have government-funded reimbursement systems that subsidize specific healthcare services and treatments for citizens, depending on each Member State's healthcare policy. Article 7 of Directive 2011/24/EU addresses reimbursement of cross-border healthcare services within the EU (11). It establishes that when a patient receives healthcare in another Member State, the health insurance system of the patient's country of affiliation must reimburse the treatment costs if the treatment is covered under its public health system. However, reimbursement is subject to conditions such as the treatment being necessary, aligned with the patient's healthcare needs, and meeting the country of affiliation's quality and safety standards. For cross-border telemedicine, telemedicine services offered by providers in one EU country are eligible for reimbursement by the patient's health insurance in their country of affiliation if these criteria are met. Member States may also require prior authorization for specific telemedicine service types to ensure alignment with national healthcare regulations. While this baseline exists, reimbursement for cross-border telemedicine services remains unclear and difficult to navigate for patients and health professionals, as criteria may vary across countries and may not be part of basic benefit packages (62).

Funding support implementation and transparent reimbursement rules are critical for scalable, sustainable cross-border telemedicine services. For example, Troschke et al., showed EU funding's important role in establishing cross-country collaboration and ensuring health professionals are compensated. Ideally, reimbursement rules should align with standards that ensure fair compensation for patients and service providers (63). These rules should be publicly accessible so patients and providers understand rights and responsibilities before service use or provision. Schemes should incorporate fraud control mechanisms, monitoring, and clearly defined penalties for non-compliance (64).

Cost-benefit assessment frameworks, drawn from existing models like health technology assessments, should be integrated (65). Such frameworks apply scientific evidence to evaluate effectiveness, quality, and added value for citizens. Pilots could test critical telemedicine services and reimbursement schemes across the EU, especially in medical areas with specialist shortages, demonstrating value and feasibility before widespread rollout. This could garner political support essential for adoption and implementation of cross-border services.

#### 4.2.4 Clinical & cultural

The clinical and cultural layer plays a critical role in cross-border telemedicine success or failure, directly affecting healthcare delivery, professional engagement, and patient outcomes. Existing referral systems enable cross-border telemedicine, such as planned care mobility schemes or border region practices where HP have developed referral systems that can be leveraged (60, 66). Another example is the ERN, which enable cross-border collaboration among specialized HP for rare, low-prevalence, complex diseases requiring highly specialized care. By connecting scattered information and expertise across EU, the ERN ensure high care standards for pan-European challenges (61, 67).

However, several challenges persist, including compatibility and comparability of clinical protocols, and lack of standardization in telemedicine practices, which can vary significantly across borders. This can lead to issues with trust, communication difficulties among HP, and reluctance to cooperate. While some organizations may have developed such protocols with specific partners in existing joint cross-border initiatives, this often limits its scalability as it only reflects an agreement based on the shared vision of those institutions (37, 40, 49, 53). Thus, it could be beneficial to start with pilot projects in clinical areas where international collaboration already exists for establishing clinical protocols, such as those in cancer treatment, or building on existing cross-border practices, as discussed in this article, to gather valuable insights for boarder implementation. However, this should also be accompanied by political and financial support to ensure its implementation and future adoption.

Clinical governance in a cross-border setting is also key for establishing trust, as the responsibility for the patient care may be unclear when multiple countries are involved. This also intertwines with the liability issues addressed in the legal and regulatory layer (37, 53). Moreover, the limited evidence surrounding the effectiveness and added-value of cross-border telemedicine practices may cause HP to hesitate in prescribing such services, especially when there is uncertainty about the potential outcomes. The absence of adequate training may also add to this reluctancy in adopting telemedicine (both locally and cross-border). Additionally, concerns over malpractices, with HP fearing exposure to legal risks, is another significant factor to be considered, particularly when navigating unfamiliar cross-border regulations and standards (49, 53). Thus, clear guidelines and training is of essence to address these challenges, together with tailored legal frameworks.

Cultural factors also play a role in the acceptance of cross-border services. For instance, there is vastly multicultural environment, with multiple languages, and not all citizens and health professionals have a functional understanding of a widely used language such as English. This can hinder effective communication and trust-building, and can lead to misunderstandings arising from language differences, which in turn can result in incorrect misinterpretation of symptoms, incorrect diagnoses and inappropriate treatment recommendations (53). Troschke et al. identified this as an important enabler to the availability of translators, but this involves additional costs and difficulties in covering multiple processes (35). Contrarily, automated translation tools or multilingual telemedicine platforms can help mitigate language barriers to some extent. However, depending solely on these technologies can be problematic, as they may fail to capture the nuances of medical terminology or the cultural context, leading to potential misinterpretations. The use of semantic standards in structured reports could help to mitigate translations issues between countries. However, this also requires significant investment and human resources to ensure highly structure data entry and reporting. Additionally, while the implementing acts under the EHDS Regulation will offer guidance on semantic terminology, achieving broad consensus on the adoption of common standards is not always feasible (10, 53, 68, 69).

Understanding cultural beliefs and expectations is also crucial for HP to provide patient-centered care, although one may argue that this has implications in both local and cross-border healthcare contexts (53). Tailoring telemedicine services to align with cultural norms can enhance patient satisfaction and adherence to treatment plans. Communication styles may also differ across cultures, with some preferring directness against a more indirect communication. Telemedicine platforms must accommodate these preferences to ensure effective communication, especially as non-verbal cues like body language and facial expressions can be limited in virtual consultations.

Establishing comprehensive educational, training and awareness programs for both HP and patients would be key to ensure little-to-no information is lost in translation. These trainings should also include culturally sensitive communication and integrating features that enhance interaction, such as clear visuals or visual aids, for improving telemedicine effectiveness. Moreover, HP should be trained in data entry and monitorization on telemedicine applications to reduce knowledge gaps amongst countries. On the other hand, patients should receive easily accessible guidance on how they can leverage these applications to promote user acceptance (70). Indeed, digital health literacy plays a crucial role in the success and acceptance of telemedicine applications; it involves the ability to use digital tools to access, manage, and evaluate health information, ensuring equal access to health services in an increasingly technology-driven society. Despite this, challenges arise, particularly for elderly populations, people in rural areas, or those with low socioeconomic status. To ensure equitable access, digital literacy programs must be implemented alongside the development of user-friendly technologies. The goal is to close the digital divide, enabling everyone, regardless of their technological skills or background, to benefit from digital health services, to prevent potential exclusion due to lack of literacy (71).

#### 4.2.5 Technical & semantic

While significant advancements have been made in digital healthcare infrastructure, especially in high-resource nations, inconsistencies across countries remains a significant barrier to cross-border and local telemedicine adoption. For instance, the article by Pagni et al. highlighted the substantial costs required to establish and maintain the necessary infrastructure over time (43). The integration of telemedicine tools within existing healthcare systems is another important barrier, as emphasized in the study by Peyroteo *et al*., that showed the challenges associated with integrating telemonitoring tools, such as sensors, wearables, and IoT technologies into existing healthcare infrastructures and workflows (24). This difficulty is particularly problematic in cross-border settings, where healthcare systems vary greatly in terms of technology, infrastructure and workflows, leading to fragmented data and reduced clinical value. Further illustrating these inconsistencies, a study by Máté Julesz conveys that existing EU initiatives already show uneven progress across Member States. For example, Croatia's EHR system provides multilingual clinical information, a critical feature that is not yet standard across the EU (72). The lack of data integration in EHR exacerbates these issues, hindering the exchange of comprehensive and up-to-date patient information, and ensuring that health data moves along citizens, regardless of their location.

Interoperability remains a fundamental barrier to cross-border telemedicine. Effective communication between varied healthcare systems is often hindered by varying technical standards, data formats and code systems (68, 69, 73, 74). Therefore, seamless interoperability requires the implementation of approaches that focus on maintaining high standards of data quality, such as accuracy, completeness and timeliness. These elements are key to ensure consistent monitorization and adequate diagnosis and treatment of citizens' moving across borders. Furthermore, cross-border initiatives aimed at improving data exchange should support the integration into different EHR systems, enabling HP to maintain up-to-date clinical records and patient information. This standardization will be key for telemedicine solutions to be able to be deployed effectively across different Member States. Such initiatives, including the MyHealth@EU infrastructure, offer important opportunities to securely exchange data in both directions, between the country of affiliation, and country of treatment, where the patient is receiving care (74).

As previously mentioned, the EHDS Regulation offers an opportunity to leverage MyHealth@EU to support cross-border telemedicine services, which will enable sharing health data through an existing secure infrastructure, allowing in the future to share all categories of health data under article 14. While this is encouraged, it is not mandatory for implementation. Moreover, the implementing acts under this regulation will support the EEHRxF, a machine-readable format designed to facilitate the transmission of personal electronic health data between various software applications, devices and HP. This format will enable the transmission of structured and unstructured health data, including harmonized datasets, code systems values, and technical interoperability specifications for content representation, standards and profiles, and it will serve as a key enabler to ensure that cross-border data exchange is interoperable (10). Moreover, clear definitions for EHR system conformity levels are expected, outlining their compliance with the EEHRxF, and the information required to display, to ensure the content exchanged is complete and thorough. While this regulation has the potential to reduce the diversity of healthcare standards within the EU and create a more unified framework that could support cross-border telemedicine services, the process is expected to unfold over several years. Additionally, it may not encompass all essential data types or address specific telemedicine requirements, and its application will be primarily limited to the EU.

Cybersecurity standards are another critical factor for trustworthy cross-border telemedicine services. Health data is inherently sensitive, and its exchange must be managed by systems that ensure data protection, in order to mitigate risks such as data breaches, hacking or identify theft. In line, GDPR (16) and electronic identification, authentication, and trust services (eIDAS) (75) Regulations are relevant legal frameworks for data privacy and authentication management in the EU. Cybersecurity measures are vital to protect patient data and build trust in telemedicine systems, particularly as the use of technologies like IoT devices increases the volume and complexity of health data shared across borders. Regular cybersecurity audits should be implemented to ensure the safe exchange of health data (76). Technical measures, like strong authentication, end-to-end-encryption and audit logs should be tailored to risk and legally compliant as emphasized by Pekka Ruotsalainen (41). For cross-border activities, it is essential to establish contractual agreements that guarantee equivalent data protection, enforce restrictions on secondary data use, apply anonymization or pseudonymization where applicable, and ensure system-wide certification for trust verification (40).

The establishment of requirements for telemedicine systems can be useful in building trust in cross-border telemedicine services. By setting high-quality standards, similar to those defined for medical devices, these requirements can also promote interoperability with standard EHR systems. This would ensure telemedicine platforms to exchange information seamlessly through existing healthcare infrastructures, thereby improving their reliability and efficiency in clinical practice. Furthermore, ensuring technical standardization of specifications and profiles for health data exchange is paramount. This would allow data consistency across various telemedicine platforms, and integration with local and international health systems (77).

Varying levels of access to infrastructure and bandwidth across regions also pose challenges to real-time health data sharing (39, 43). This is especially crucial for managing chronic conditions and ensuring timely interventions, highlighting the need for financial support along with adequate resources to maintain such baseline resources over time.

#### 4.2.6 Artificial intelligence as an opportunity for telemedicine

Telemedicine in itself is still a growing trend, but it has been heavily connected with the emergence of another powerful tool, AI (78–81). With the growth of Large Language Models and the broad discussion about their validity and effectiveness at diagnosing or offering valid medical advice, research into AI and healthcare has been steadily climbing (82). Nonetheless, there are careful considerations that are still required to be taken into account with the use of AI, where the EU has been attempting to strike a harmonious balance between their development and healthcare, through regulatory frameworks (83).

The AI Act [Regulation (EU) 2024/1689] (84) classifies AI tools based on their risk levels, ranging from unacceptable to minimal risk. Given that AI in healthcare can support professional decision-making or simplify certain medical procedures, it often falls under the high-risk class. For instance, AI-based medical devices for remote monitoring, regulated under Regulation (EU) 2017/745 (85), and AI for remotely triaging users (86), both require strict compliance from the providers (for the development of the AI telemedicine tool) and the deployers (responsible for implementing the solutions within the scope of telemedicine). The regulation also sets specific transparency and accountability standards that must be considered for any AI system assisting in a telemedicine operation, as well as compliance with standard data protection guidelines, such as those set out in the GDPR (16). Prioritizing transparency and explainability in AI models is also essential, ensuring that the decision-making processes and the algorithms behind them are clear to HP, while preserving human oversight (87).

Having this in mind, AI in telemedicine offers the potential to enhance patient monitoring, intelligent diagnosis, and assistance. It can improve healthcare delivery, automate administrative tasks, and assist physicians in decision-making (19). Artificial intelligence in itself could facilitate the implementation of cross-border data sharing, as its use could improve the access to real-world health data and their integration within health information systems and medical devices for remote monitoring, as will be assessed by the SHAIPED European project (88). In addition, AI can also enhance structured data reporting that could help to minimize the barriers mentioned above, by supporting HP in improving coding accuracy, clinician efficiency, and understanding. It can facilitate transcoding for cross-border purposes, allowing the exchange of translated information into the local language (81, 89), following similar procedures as those applied in MyHealth@EU (90). Furthermore, AI could also aid in mitigating certain barriers in telemedicine procedures, such as telesurgery by minimizing delays in input communications through error correction algorithms, or more importantly allowing the integration with surgical medical devices to allow virtual training applications (91).

However, AI must align with clinical practices and consider factors such as trustworthiness, reproducibility, usability, availability, transparency, and cost for optimal effectiveness. Thus, AI has emerged primarily of interest in four key areas: 1) patient monitorization at a distance, 2) use of intelligent decision-making and cooperative diagnosis, and 3) processing data collaboratively, simplifying the amount of time doctors spend utilizing EHR and 4) simplifying semantic mapping, that simplifies language barriers and document generation in the native language of HP (92).

Despite all of this, in order to implement AI-enabled telemedicine several challenges are still needed to be overcome, such as (92, 93):

i) Access to technology and capable infrastructure: a significant hurdle is the limited access to essential technology, namely when healthcare is not financed properly. AI-driven telemedicine relies on capable internet connections, mobile devices, and large data processing facilities, which may be scarce in under-resourced areas, complicating the implementation and utilization of the service.
ii) Training and change management: new training requirements and change management processes to introduce new technologies, combined with the shortage of HP is complex, especially considering the excess hours and the strain placed on healthcare locations. AI-enabled telemedicine may require specific training or specialized skills for system development, operation, and maintenance.
iii) Concerns regarding data usage and privacy: the AI Act lays out the required guidelines to ensure data input into AI tools is secure, but it is always a source of concern. Protecting patient data is a critical issue. The exchange and storage of sensitive information in AI-powered telemedicine systems must be secure, but many resource-limited areas lack the infrastructure to support adequate data protection, making these systems vulnerable to breaches.
iv) Lack of funding: lastly, the financial burden of establishing and maintaining AI-enabled telemedicine can be onerous, especially in low-resource settings.

With the publication of targeted regulations and guidelines to help providers shape their products, this area is foreseen to grow in expression over the coming years.

## 5 Conclusions and future perspectives

This study aimed to offer a complete overview of the current landscape of telemedicine solutions and covered a wide range of studies that were performed in different care settings. Telemedicine has been an emerging field and undeniably revolutionized access to healthcare by facilitating easier disease monitoring, simplifying patient access to specialized medical help, and improving therapeutic outcomes. The ability to connect patients with HP remotely has transformed the way medical care is delivered, especially for those in underserved or rural regions. Cross-border telemedicine has emerged as a crucial component of this evolution, allowing patients to receive expert opinions and treatments that were once limited by geographic and resource constraints, contributing towards a novel interconnected world. Through this approach, the integration of multi-expert platforms enables a collaborative approach to patient care, drawing on the expertise of specialists from around the world and offering a powerful solution for managing complex or rare diseases, and drastically reducing barriers to getting the best treatment options.

While challenges remain, such as legal and regulatory uncertainties, limited financial resources, diverse clinical practices and cultural differences, technical limitations and concerns about data security and privacy, these obstacles present opportunities for innovation and improvement. Moreover, significant progress has also been made as showed in this review, which can be used as a crucial baseline for the development of collaborative cross-border solutions aimed at minimizing existing barriers that hinder the widespread deployment of cross-border telemedicine services. Overcoming these challenges will be essential in fully realizing the potential of telemedicine, particularly in the context of cross-border care. Critical initiatives, such as the EHDS Regulation that will revolutionize the access to health data by citizens, giving them full control over their information and allowing them greater mobility within the EU, building on what was initiated by the groundbreaking initiative MyHealth@EU, will effectively work towards seamless, secure, and interoperable health data exchange across borders, laying the foundation for a more integrated, collaborative, and patient-centered healthcare ecosystem.

By analyzing these studies and contextualizing them within the regulatory framework under the EHDS Regulation, potential novel applications and policy recommendations can be thoroughly informed on the key benefits and challenges essential to support the implementation of cross-border telemedicine. While the EU has introduced important key regulatory frameworks, these do not cover all requirements necessary for scalable and reliable implementation of cross-border telemedicine programs, and should be further complemented by other key legal frameworks and guidance.

This review can serve as a key resource for future policy work at EU level regarding the implementation of cross-border telemedicine, as policymakers can potentially leverage the analysis of enablers and barriers to better target upcoming regulations and strategies to streamline the adoption of local and cross-border telemedicine services. Furthermore, the information from this review can enable the development of specific guidelines that consider the intrinsic requirements of cross-border telemedicine based on five important operational areas (legal and regulatory, organizational, financial, clinical & culture, technical & semantic). Future EU policy should focus on creating incentives for Member States to better integrate and expand telemedicine solutions, while also encouraging cross-country collaboration through multi-specialist networks. The use cases presented in this review illustrate the advantages of cross-border telemedicine services, especially in light of the overburdened healthcare systems and lack of resources, while underscoring the opportunity to build on the momentum generated by the EHDS Regulation.

These improve healthcare access, clinical outcomes, enabling faster, informed decision-making. Healthcare future is global, interconnected, delivering high-quality care regardless of location.

Despite the identification of several enablers and barriers for the implementation of cross-border telemedicine, some limitations of this review should be acknowledged and addressed in future work. First, a more detailed legal analysis of the regulatory instruments governing telemedicine is needed, considering differences between Member States as well as provisions related to data protection and data use more broadly. Second, further examination of the technologies used in telemedicine (including AI), along with the relevant interoperability frameworks and semantic standards, would help to better understand the potential evolution of telemedicine in the coming years. Finally, issues related to security, trust anchors, and the robustness of patient authentication mechanisms should be investigated to fully understand the obstacles affecting the implementation of cross-border telemedicine services.
